# Discovery and Expansion of Gene Modules by Seeking Isolated Groups in a Random Graph Process

**DOI:** 10.1371/journal.pone.0003358

**Published:** 2008-10-09

**Authors:** Jochen Brumm, Elizabeth Conibear, Wyeth W. Wasserman, Jennifer Bryan

**Affiliations:** 1 Department of Statistics, University of British Columbia, Vancouver, British Columbia, Canada; 2 Centre for Molecular Medicine and Therapeutics / Child and Family Research Institute, University of British Columbia, Vancouver, British Columbia, Canada; 3 Department of Medical Genetics, University of British Columbia, Vancouver, British Columbia, Canada; 4 Michael Smith Laboratories, University of British Columbia, Vancouver, British Columbia, Canada; Tel Aviv University, Israel

## Abstract

**Background:**

A central problem in systems biology research is the identification and extension of biological modules–groups of genes or proteins participating in a common cellular process or physical complex. As a result, there is a persistent need for practical, principled methods to infer the modular organization of genes from genome-scale data.

**Results:**

We introduce a novel approach for the identification of modules based on the persistence of isolated gene groups within an evolving graph process. First, the underlying genomic data is summarized in the form of ranked gene–gene relationships, thereby accommodating studies that quantify the relevant biological relationship directly or indirectly. Then, the observed gene–gene relationship ranks are viewed as the outcome of a random graph process and candidate modules are given by the identifiable subgraphs that arise during this process. An isolation index is computed for each module, which quantifies the statistical significance of its survival time.

**Conclusions:**

The Miso (module isolation) method predicts gene modules from genomic data and the associated isolation index provides a module-specific measure of confidence. Improving on existing alternative, such as graph clustering and the global pruning of dendrograms, this index offers two intuitively appealing features: (1) the score is module-specific; and (2) different choices of threshold correlate logically with the resulting performance, i.e. a stringent cutoff yields high quality predictions, but low sensitivity. Through the analysis of yeast phenotype data, the Miso method is shown to outperform existing alternatives, in terms of the specificity and sensitivity of its predictions.

## Introduction

Much of systems biology research aims to identify biologically meaningful relationships between genes or their products, such as protein-protein interactions or co-membership in a biological pathway. This undertaking can be viewed as moving from the “parts lists” produced by genome sequencing projects to the assembly instructions for a complex system.

The combination of entities and their relationships is often described as a network, which can represent diverse biological systems such as cellular or signal transduction pathways [1], [2]. A common assumption made in the analysis of networks is the existence of biologically defined subnetworks commonly referred to as modules. Examples of such a module are a protein complex or a gene expression regulon.

Quantitative data from diverse genome-scale experiments can be exploited for the identification of new modules and the expansion of known modules. Correlation of expression levels or, more relevant to this study, loss of function phenotypes across multiple conditions provides an indirect measure of gene–gene relationship. Other assays such as yeast two-hybrid or genetic interaction screens using double knockouts provide direct measures of these relationships. Early approaches to such studies were limited by a binary representation of the observations, but increasingly more powerful analysis is enabled by quantitative readouts [3]–[5].

While the quantitative data can be highly reproducible and informative, identifying the relevant functional relationships can still be a challenge. In noisy data there is great risk of predicting a spurious relationship between any pair of genes. An analytical approach based on modules, however, moves the focus from individual to connected sets of relationships. To invoke a concept from social network analysis, there is greater evidence for a relationship reinforced by common associations than for an individual, seemingly strong pairing. This principle is the basis for many algorithmic approaches for network identification [6]–[8].

The two main paradigms for module finding utilize different representations of the relationships: (i) a graph is obtained by applying a global threshold to the relationship data; or (ii) a hierarchy such as a dendrogram (or tree) is produced by a clustering algorithm. In graph-based approaches, nodes represent genes and edges represent relationships. A ‘threshold graph’ is obtained from continuous relational data by classifying all pairs with similarity above the chosen threshold as related, and all other pairs as not related. The graph is subsequently processed, for instance based on the density of intra-group relationships, to produce candidate modules. In tree-based approaches, genes appear as leaves connected by branches, where branch height corresponds to some measure of relationship strength. Gene groups are obtained by pruning the tree, often by invoking a global height threshold.

In both approaches, the specification of a global threshold is fundamentally limiting. Modules in genomic data can be dissimilar: they can vary greatly in size, in internal cohesion (how ‘related’ two genes within a module are) and external isolation (how ‘unrelated’ the genes in the module are to genes in other modules). No single threshold graph or pruning of a tree reveals all of the modules in a heterogeneous biological system. Both methods are limited in their ability to perform well for the simultaneous analysis of all modules and are extremely sensitive to the selection of the threshold parameter.

We develop a novel approach for the detection of modules in relational genomic data. Our approach is fundamentally based on the *ranking of the relationships* between genes. Viewed in terms of the graph paradigm introduced above, we work with the entire sequence of threshold graphs that result from sliding the global threshold from stringent to permissive. Modules in this sequence of graphs are identified as groups that appear and persist as cohesive subgraphs. This approach for the detection of module isolation, which we refer to as the Miso method, permits the identification of modules with differing internal cohesion and determines the statistical significance of each candidate module. Extending a theoretical method introduced by Ling [9], the Miso method can also be used to score clusters in any single linkage dendrogram. In application to two collections of yeast mutant data [10], [11], we show that our method successfully identifies known modules. Furthermore, our method predicted a new module which was subsequently experimentally confirmed as a novel protein complex [11]. A comparative study establishes that the Miso method performs very well relative to several alternative methods based on the post-processing of threshold graphs or dendrograms. Additionally, this comparison underscores the practical advantage offered by the tuning parameter of the Miso method. Its natural interpretation as a measure of stringency provides external guidance when selecting a value appropriate for a specific application and, more generally, implies a predictable relationship between its value and classical measures of performance. We developed a Cytoscape [12] plug-in and R [13] code to make the methods available to the community.

## Results

### Dissimilar biological modules in relational data

**Figure 1:**
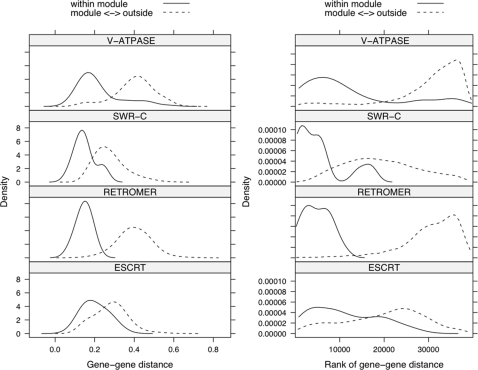
No global threshold exists for the simultaneous recovery of all network modules. Smoothed histograms of the observed relationships in which both genes lie within (solid lines) and exactly one gene lies within (dashed lines) four well-characterized modules addressed by the yeast vesicle transport data [11]. Euclidean distance is presented on the original scale and a rank scale in the left and right panels, respectively The plots depict the heterogeneity in the internal cohesion and external isolation of biological modules. Within the left and right panels, all plots have a common Y-axis. i.e. identical limits and tick marks.

We assume that genomics data arrives in the form of ranked pairwise relationship scores (e.g. derived from Euclidean distance or correlation). While such data can be generated by many approaches and take many forms, for the purpose of this report we analyze only yeast mutant phenotype data, including a study aimed at the global identification of endosomal transport factors [11] (full description to follow below). In Figure 1 we present information on four well-characterized modules, chiefly protein complexes implicated in vesicle transport in yeast. We compare the strength of relationships in which both genes belong to a module to those in which exactly one gene belongs to the module. We present both ranked relationships and the associated underlying continuous association measures. For all these modules the intra-module relations generally are stronger than the extra-module relations. However, the threshold that provides the best modular distinction varies noticeably between modules. Summarizing, there is no global threshold that is ideal for the recovery of all network modules.

### The graph process captures evolving relationships across a spectrum of thresholds

**Figure 2:**
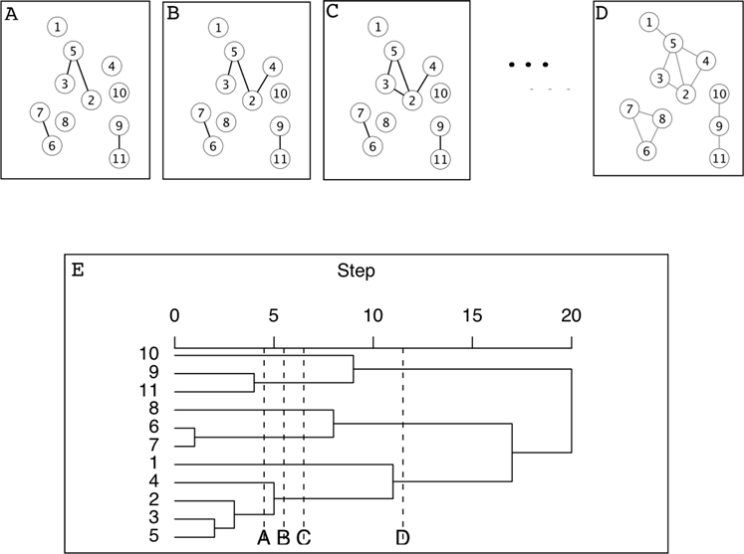
Illustration of a graph process and the birth and death of identifiable subgraphs. A graph process proceeds by sequentially adding edges in rank order. When two subgraphs, defined here as singly connected components, are joined, two candidate modules ‘die’ and a new candidate module is born. Survival time is defined as the number of edges added in the graph process between birth and death. We show steps 4,5,6 and 11 here in panels A, B, C and D, respectively. In B a ‘between’ edge joins subgraphs (2,3,5) and (4) into a new subgraph. In C, a ‘within’ edge is placed which does not affect subgraph membership. In D, the subgraph born in B dies resulting in a survival time of 11−6 = 5. Panel E provides the corresponding single linkage dendrogram. Note that the height of cluster merge events corresponds exactly to death and birth events of subgraphs.

A graph process is a useful representation of pairwise relationships. In contrast to a single graph, a graph process is an ordered set of graphs generated by incrementing a parameter. Conceptually within the process this parameter can be thought of as time. The process is initiated with a graph that has all genes but no edges. The next graph is obtained by placing an edge between the pair of genes with the strongest relationship score, i.e. rank 1. Subsequent edges are added in the order of gene-gene relationship scores, i.e. ranks 2, 3, …, as illustrated in Figure 2. This results in a sequence of graphs that starts with an empty graph and ends with a complete graph. When a global threshold (i.e. one value of the parameter) is applied to relational data, the entire analysis is based merely on a single graph.

### Candidate modules are subgraphs of significant persistence

It is our thesis that modules will appear and persist within this graph process for a period of time as identifiable subgraphs.

**Figure 3:**
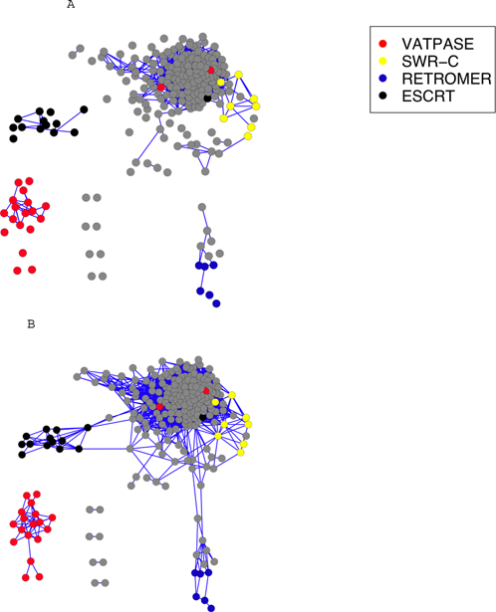
The observed graph process for yeast vesicle transport data at step 2000 (a) and 5000 (b). Node color corresponds to module membership; unannotated genes appear in grey. Specific identifiable subgraphs in panel a) have been incorporated into larger subgraphs in panel b).

The most straight-forward, identifiable subgraphs are the ‘singly connected’ components that arise during the graph process. These subgraphs have the defining property that every gene pair is linked by a sequence of edges within the subgraph and no edge connects to this subgraph from the outside. Figure 3 presents an example of a graph process produced by the ranked yeast vesicle transport data. The emergence (Figure 3a) and disappearance (Figure 3b) of subgraphs corresponding to modules (here, complexes and/or pathways associated with the vesicle transport system) can be observed. The set of all singly connected components appearing in the graph process can be enumerated and form a set of candidate modules.

### Figure of merit for candidate modules based on survival time

To facilitate interpretation, the candidate modules must be assessed with a quantitative measure of significance. Such a score ranks candidates for expensive validation studies and provides an objective measure of confidence. Our measure of significance for a candidate module is based upon the length of time it survives within the graph process as an identifiable subgraph. We say a subgraph is born when the associated set of nodes first becomes singly connected and dies upon the placement of the first edge connecting a node in the subgraph to a node outside the subgraph. The survival time is the difference between death and birth. Figure 2 illustrates birth, death, and survival of candidate modules in a simple example.

Following Ling [9], we assess the statistical significance of an observed survival time by comparing it to the distribution of survival times in a randomly evolving graph process. At the birth of a specific candidate module, each remaining edge can be classified based on the two associated nodes; the edge is ‘within’ (both nodes in the candidate), ‘between’ (exactly one node in the candidate), or ‘outside’ (neither node in the candidate). The death of the candidate module occurs upon the placement of the first ‘between’ edge. The distribution of this waiting time under random evolution is easily obtained and, therefore, we can compute a p-value for the observed survival time. Intuitively, this method assumes that ‘within’ edges typically arrive before ‘between’ edges and that biological modules will often appear as identifiable subgraphs that enjoy unusually long survival times. We refer to this p-value as an isolation index.

### Augmenting the list of candidate modules: removing high leverage edges

Noise in the data can lead to the premature placement of edges between genes belonging to distinct biological modules, which violates the assumption that ‘within’ edges arrive before ‘between’ edges. Such noise could arise from the limitations of the experimental assay or from true biological heterogeneity (e.g. a gene belongs to multiple modules). In our procedure, where candidate modules are singly connected subgraphs, such errors in edge order can affect survival times and even the composition of the list of candidate modules. Our method could fail to detect a true biological module if its survival time is truncated or if, when it first emerges, it is already embedded within some larger group of genes. These two problems arise when a mistimed edge arrives after or before, respectively, the birth of the module.

To make our Miso procedure robust to this sort of error, we extend it by considering the impact of high-leverage edges, i.e. the ‘between’ edges whose placement cause the death of a candidate module. To mitigate the effect of these high leverage edges that hit a module after birth, we compute the waiting time and associated p-value for the arrival of the *k*-th ‘between’ edge, for *k = 1,2, …, K*, and define the extended *k*-isolation index as the minimum of these *K* p-values. To reduce the impact of edges that hit a module before birth, we consider parallel graph processes in which each individual high-leverage edge is postponed until the end. We extract candidate modules and associated p-values from these processes using the procedures described above. We form the list of candidate modules obtained from all one- and two-edge removed processes.

To introduce the nomenclature that follows - the number of allowed mistimed edges before and after birth are given in parentheses. For example, Miso(0,1) refers to the modules that are not hit before birth, and for which isolation is assessed until the first edge hits after birth; Miso(2,6) refers to the isolated modules that were hit at most twice before birth and tracked until at most six hits have occurred after birth.

### Relationship to single linkage clustering

Our approach, in which candidate modules are singly connected components, is related to single linkage hierarchical clustering. The candidate modules identified by Miso(0,1) are exactly the clusters arising in the dendrogram. Therefore one broadly useful application of our method is the selection of significantly isolated clusters from single linkage clustering (see Figure 2A in [11]). While dendrograms are a useful representation of single linkage clustering, clusters that are significantly *k*-isolated with *k>1* may not be detectable by visual inspection. Candidate modules detected via the removal of one or more high leverage edges may not even appear as clusters in the dendrogram.

### Analysis of vesicle transport and DNA damage response in yeast

For the model organism *S.cerevisiae* the research community has created a collection of modified strains in which each member of a panel has a distinct gene disabled [14], [15]. Using an appropriate assay, the phenotype of each strain in the panel is measured under a set of conditions. It is anticipated that for two genes within a module their respective mutants will display similar properties. We apply our methods to yeast mutant phenotype studies of two important systems - vesicle transport [11] and DNA damage response [10]. The modules within the vesicle transport system are well annotated, making this set suitable for the evaluation of our analytical method. Although the modules are less deeply annotated, we present an analysis of the DNA damage data as an independent validation.

We first applied our methods to a data set exploring vesicle transport. In eukaryotic cells, the directed movement of substances in membrane-bound sacs (vesicles) within the cell is called vesicle transport. Vesicle traffic is regulated by protein modules that select cargo for incorporation into a forming vesicle and direct vesicle docking and fusion with the appropriate target membrane. The modules tend to be conserved between species, thus knowledge generated in studies of yeast can reveal the biochemical mechanisms by which defects in protein and lipid trafficking contribute to human disease.

Quantitative phenotypes were obtained under 14 conditions for 279 genes that displayed a strong phenotype in an initial, independent genome-wide screen. The 279 genes include 137 genes known to belong to 25 modules. In the analysis reported in [11], we used the Miso(0,1) method with great success and the key results are displayed on top of a dendrogram. For example, the two largest candidate modules correspond almost exactly to two previously known modules–namely, the protein pump V-ATPase and the ESCRT subcomplexes (Table 1). Another high-scoring candidate module (“55-68”) was subsequently validated in prospective experiments that confirmed a predicted protein-protein interaction. Here, in addition to the most conservative implementation [Miso(0,1), Table 1], we also apply our method in a more aggressive form [Miso(2,6), Table 2] to the vesicle transport data. We find that 78% [Miso(0,1)] and 63% [Miso(2,6)] of the predicted within-module relationships are, in fact, ‘true’, i.e. are implied by the prior knowledge, and that 48% [Miso(0,1)] and 53% [Miso(2,6)] of true relationships are successfully predicted. The Miso methods perform as well or, arguably, better than published alternatives at recovering and expanding modules in the yeast vesicle transport system (detailed further below).

**Table 1:** Results for the Miso(0,1) method for the vesicle transport data.

Candidate module	Size	Birth	Death	p-value	Composition
V-ATPase	18	4314	7922	6.82E-228	V-ATPase (18)
ESCRT	13	4348	4506	1.52E-05	ESCRT(13)
Retromer (I)	10	3224	3530	9.76E-09	Retromer(4),PI3K(2),ClassD *VPS*(1), ClassA/D *VPS* (1)
COG/YPT6	9	1252	1468	1.46E-04	COG(4),YPT6(4),ARF(1)
SWR-C	6	1073	1524	5.56E-07	SWR-C(6)
INO80	4	1413	2326	3.95E-10	INO80(2)
55-68	3	463	2072	1.51E-13	55-68(2),ClassD *VPS*(1) validated
ClassB	3	6451	7599	3.23E-11	ClassB *VPS* (3)
PI3KC	2	12208	21651	1.10E-84	PI3K(2)
ClassD	2	2644	6344	4.16E-23	ClassD *VPS* (2)
GARP	2	1029	2923	1.92E-10	GARP(2)
Retromer(II)	2	130	1844	2.70E-07	Retromer(2)

The table shows the composition of significantly isolated candidate modules (Bonferroni corrected p-value less than 0.05), and is a subset of the results in [11]. The “birth” column gives the step in the graph process when the module is first connected, the “death” column gives the first time an edge from outside hits the module, the p-value is computed with Miso(0,1). Most candidate modules can clearly be associated with a protein complex. The candidate 55-68 contains a relationship validated in [11]. Omitted are a candidate module of size 3 and 7 of size 2 with unknown annotations.

**Table 2:** Candidate modules derived from the vesicle transport data using the Miso(2,6) method with a Bonferroni-corrected p-value cutoff of 0.05.

Candidate module	Size	Remarks
V-ATPase	21	V-ATPase(18),ClassC *VPS*
ESCRT	13	ESCRT(13)
Retromer	12	Retromer(6),PI3KC(2)
SWR-C	11	SWR-C (8)
YPT/COG	9	YPT(4),COG(4),ARF(1)
55-68	4	55-68(2),ClassD *VPS*
EE	3	EE(2)
Glycosyl	3	Glycosyl(2)
PI3KC	3	PI3KC(2),ClassC *VPS*(1)
ClassD	3	ClassD *VPS*(3)
ClassB	3	ClassB *VPS*(3)
DNA	3	SWR-C(1), RSC(2)
GARP	2	GARP(2)

We then applied our methods to the DNA damage response phenotype data described in [10]. DNA damage response pathways are relevant for cancer in humans, both for prevention and treatment. In [10] the authors analyze the phenotypic response of 140 deletion mutant strains in 36 conditions related to exposure to DNA damaging agents. From the average linkage dendrogram, interpreted in light of expert knowledge, the authors selectively identified the following six functional groups containing 23 genes:

- C1: NER (RAD2, RAD4, RAD10, RAD14, and RAD1)
- C2: error-prone TLS (REV1 and REV3);
- C3: PRR (RAD6, RAD18, and RAD5);
- C4: homologous recombination (RAD57, RAD51, and RAD54);
- C5: cell-cycle checkpoint control (RAD9, RAD24, RAD17, DDC1, and MEC3)
- C6: (SHU2, SHU1, CSM2, MPH1, and PSY3).

We recover these hand-picked modules in an objective fashion using our methods (Tables 3 and 4). The conservative Miso(0,1) output contains modules C1 and C2 perfectly, with partial recovery of C3 (2 of 3 predicted; no additional predictions), C4 (2 of 3; 2 additional genes included) and C5 (2 of 5; no additional). Two additional modules of 3 genes each were predicted. Analysis with Miso (2,6) recovers C1, C5 and C6 perfectly. Compared to the Miso(0,1) results, C3 is unchanged and both C2 and C4 have one additional gene. In addition, the Miso(2,6) method predicts only one other candidate module, with the noteworthy property that 4 out of the 5 genes are known to be involved in DNA repair.

**Table 3:** Candidate modules derived from the DNA damage data using the Miso(0,1) method with a Bonferroni-corrected p-value cutoff of 0.05.

Candidate module	Size	Birth	Death	p-value	Remarks
RAD4, RAD2, RAD10, RAD14, RAD1	5	2233	6760	3.68E-145	5/5 from C1
RAD18, RAD5	2	995	5525	1.40E-52	2/3 from C3
MMS4P, YBR099C, MUS81	3	364	2838	3.73E-41	Not on list
REV1,REV3	2	13	2862	6.06E-31	2/2 from C2
RAD9, RAD24	2	252	795	1.34E-06	2/5 from C5
LTE1,BCK1,CLA4	3	219	490	4.03E-05	Not on list
RAD57,RAD55, RAD51, HPR5	4	162	353	7.64E-05	2/3 from C4

**Table 4:** Candidate modules derived from the DNA damage data using the Miso(2,6) method with a Bonferroni-corrected p-value cutoff of 0.05.

Candidate module	Size	Remarks
RAD4,RAD2,RAD10,RAD14,RAD1	5	5/5 from C1
RAD5,RAD18	2	2/3 from C3
REV1,REV3,RAD23	3	2/2 from C2
RAD59,MMS4P,YBR099C,PPH3,MUS81,SAE2	6	4/5 DNA Repair
SHU2,SHU1,CSM2,MPH1,PSY3	5	5/5 from C6
RAD9,RAD24,MEC3,RAD17,DDC1	5	5/5 from C5
RAD51,RAD57,RAD55,RTT101,HPR5	5	2/3 from C4

### Comparison of methods

**Figure 4:**
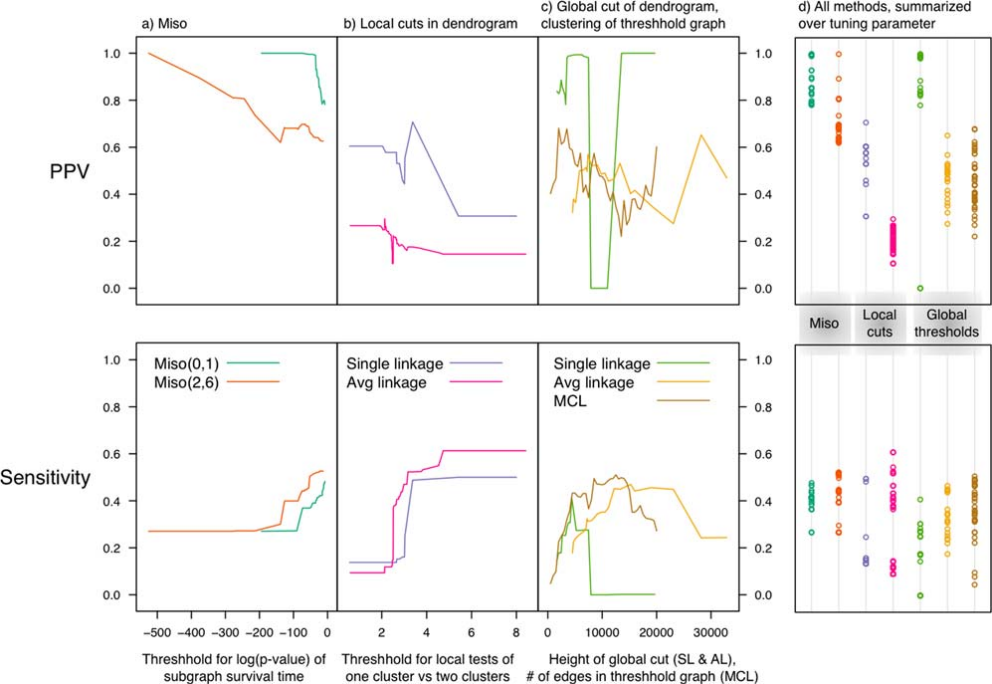
Relative performance of module detection methods applied to yeast vesicle transport data. Displayed are the PPV (top row) and sensitivity (bottom row). The horizontal axes correspond to the tuning parameters specific to each class of methods; see Materials and methods. For the Miso methods in column a), the tuning parameter is the threshold applied to module-specific p-values. For the local cuts in column b) the tuning parameter is the rejection value for the Duda-Hart test statistic. For the global methods in column c), the tuning parameter corresponds to a step in the graph process. The puzzling behavior of the single linkage PPV curve in column c) results from a late joining gene pair corresponding to a true biological relationship. Column d) summarizes the range of PPV and sensitivity values.

To assess the relative performance of the Miso method, we applied it along with alternative methods to the vesicle transport data (Figure 4). The DNA damage response data is less suitable for comparative analysis due to the sparse annotations; the results are given for completeness in Figure S1, but will not be discussed in detail here. We selected representatives from the two broad categories described above: graph-based and dendrogram-based methods. We used MCL [8] as the representative graph clustering procedure because it performed well in a benchmark test [16]. For the identification of modules within a dendrogram, we consider both global cuts, including the one suggested by the gap statistic [17], and local cuts. Objective local cuts, although not commonly used in genomics, are included because they are the most similar conceptually to the Miso method. Based on the work of Milligan and Cooper [18], we employ the local cut criterion introduced by Duda and Hart [19].

All of these procedures must be supplied with a tuning parameter to return a list of candidate modules. The tuning parameters of the methods we study are conceptually very different. For the Miso method, the tuning parameter is a p-value cutoff and therefore is a measure of stringency (i.e. the closer the parameter is to zero the higher the positive predictive value). For none of the other methods is there such a simple relationship between the tuning parameters and the performance of the method. MCL requires the specification of a similarity threshold which, in effect, corresponds to the selection of a single threshold graph. MCL proceeds to identify candidate modules as dense subgraphs within the selected graph. In the context of a dendrogram, a global cut across a tree partitions the genes into clusters which are the candidate modules. This global cut can be viewed either as the selection of a similarity threshold, as in MCL, or more relevant in actual practice, as specifying the number of clusters. Local cuts applied to a dendrogram are implemented in a bottom-up manner to determine when to merge clusters based on their lack of separation.

Based on existing annotation of modules associated with vesicle transport, any relationship (edge) between two members of the same module is considered “true”, all other relationships “false”. Two metrics are computed to quantify performance. We use the positive predictive value (PPV), which gives the rate of true positive predictions among all predictions, and the sensitivity, which gives the proportion of true relationships predicted. Biological knowledge is incomplete, therefore a portion of the “false” predictions will be true–i.e. the reported PPV measures are conservative. Groups of greater than 50 genes were not considered as valid candidate modules, since the true biological modules of interest are of much smaller size.

The results for our comparative study, given in Figure 4, show that the Miso methods are the best with respect to PPV and match the performance of the other methods with regards to sensitivity. In Figure 4a), the Miso methods perform as expected with Miso(0,1) making a smaller number of higher quality predictions relative to Miso(2,6). The local cut methods perform uniformly worse with respect to PPV and exhibit maximum sensitivities that are comparable to those of the Miso methods (Figure 4b). The most striking finding for the global cut methods is the volatile relationship between the tuning parameters and performance, especially for PPV (Figure 4c). This volatility demonstrates the importance of the tuning parameter as well as the difficulty of choosing its optimal value, particularly in the absence of known annotations.

## Discussion

Based on the analysis of graph processes, we have introduced a novel method for the identification of biological modules in ranked relational data. Building on a theoretical foundation from Ling [9], the Miso methods accommodate the heterogeneity and noise that is inherent to genomic data and detect modules that vary widely in size, external isolation and internal cohesion. An objective measure of confidence–a p-value–is assigned to each candidate module, prioritizing candidates for further study. Because the isolation index is a measure of stringency, it is particularly attractive for applications in which there is little or no prior biological knowledge to guide the selection of tuning parameters.

In the ongoing effort to identify modules from genomic data, the most dominant methodological approaches are based on one of two representations of the data: graphs and hierarchical clusterings (dendrograms). Regardless of the analytical paradigm, a key challenge is to overcome the combined effect of biological heterogeneity and experimental variability. The distinctive, individual properties of real biological modules generally imply that there is no universal ‘signature’ that would enable module detection based on thresholding relationship strength or, by extension, some related summary measure. This reveals, therefore, a fundamental limitation of methods based on threshold graphs or the global pruning of dendrograms. In the presence of diverse modules, the Miso methods are better able to perform well for many modules simultaneously, since each candidate module is evaluated in a distinct timeframe within an evolving graph process.

It is increasingly common to address the variability in genomic relational data by using probabilistic approaches to graphs [7], [16], [20]. When analyzing a single observed graph, noise can be acknowledged by recognizing the potential error associated with each observed edge (or lack thereof). The graph process paradigm for module finding, originally introduced in [9] and adapted and extended for genomic data analysis here, offers a natural extension of the probabilistic analysis of graphs. Two specific choices in the construction of the Miso method bear further comment: the designation of singly connected subgraphs as the topological structures of interest and the use of a randomly evolving graph process as the null model. While these choices can be viewed as (over-)simplistic, we note that these specifications yield a module-specific significance score that is easily computed in closed form. In contrast, a different definition of an identifiable subgraph and/or a more complicated reference distribution would require extensive simulations to approximate the null distribution of candidate module survival times. We feel that the Miso method, in its simplicity, represents an attractive compromise between conceptual elegance and practicality.

Although it has not yet been applied to the problem of gene module identification, another relevant paradigm is that of the ‘clustering tree’ of a density [21]–[23]. This approach treats observations as a sample from a (nonparametric) density, presumably having two or more modes, and equates the true underlying clusters or modules with the ‘domains of attraction’ of these modes. The interesting connection between the Miso and clustering tree methods lies in the use of a graph process or sequence. The clustering tree of a density can be obtained, or at least approximated, from the connected components arising in a sequence of graphs generated by thresholding the (estimated) data-generating density [22]. It will be interesting to include clustering tree methods in future performance studies of Miso and its extensions.

Data from genomic research tends to be very noisy. Our extensions of the original isolation index offer improved sensitivity by allowing for a few mistimed edges in the graph process. However, even the extended Miso method is sensitive to noise of the magnitude often observed in genomic data. While the quality of the predictions will remain high, the sensitivity will be diminished in datasets with less separation (due to lack of adequate assays or technical variability). We expect that with continuing development of assays lack of separation will become less of an issue, whereas the heterogeneity of complexes is inherent in the biological systems.

The study of an evolving graph process offers a promising new direction for the discovery of biological modules. Building on the groundwork laid here, an intriguing approach to the analysis of noisy relational data is to move from a model of random evolution representing the absence of modules to a generative model driven by the presence of modules. In such an approach, the probabilistic model for relational data is represented by a likelihood function that explicitly incorporates the properties of gene–gene relationships between and within modules. Such an extension provides a natural basis for solving even harder problems, such as the integration of relational data arising from distinct experiments or even different platforms.

## Materials and Methods

### Data Sources

The vesicle transport data was reported in [11]. The data is obtained by plating yeast mutant colonies in 1536-array format on nutrient media. The growth of the colonies in the presence of various chemicals or the secretion of certain proteins (as determined by biochemical assays) is measured by quantifying images by densitometry. The measurement values were preprocessed by averaging across replicates, correcting for background intensity by subtracting the values of blank spots and converting the measurement of growth or secretion into a percentage relative to the wild-type strain. In an initial, independent genome-wide screen, 279 genes that displayed a strong phenotype were selected. Quantitative phenotype measurements can be arranged in array form with the rows being the gene knockout strains and the columns the conditions, the values of this array were processed by scaling each column by its standard deviation. Indirect measures of relationships were obtained by applying Euclidean distance to the rows of the preprocessed array.

The DNA damage data was taken from [10], where the authors report an analysis of 140 genes. We take the same genes here and apply our procedure to the data using a Euclidean distance measure.

### Probabilistic model for the graph process and scoring survival times

A graph process is a representation of the ranking of quantitative, pairwise gene–gene relationships for *n* genes. Ties within the *N = n(n−1)/2* gene–gene relationship ranks were randomly ordered and the resultant ranking was held constant for all analyses reported here. To analyze an observed graph process in a probabilistic fashion, the null model assumes that all rankings of pairwise relationships are equally likely. As noted by Ling [9], this assumption does not strictly hold when the rankings are derived from a distance measure, because of the constraint that the triangle inequality imposes on pairwise distances.

As introduced above, a candidate module is a simply connected subgraph and its survival time is the difference between the rank of the edge that established the subgraph and the edge that adds a new member. Our measure of significance is the p-value for the survival time in a random graph process. Since edges are drawn without replacement, the probability of choosing a particular edge at step *t* out of all possible *N* edges is *1/(N−t)* (any of the *N-t* edges left has equal probability).

For the purpose of scoring a specific candidate module, we define ‘success’ as the placement of an edge between two genes either both within or both external to the module. We define ‘failure’ as the placement of an edge between one gene within the module and one gene external to the module; this results in the death of the candidate module. We denote the probability of failure at step *t* as *p_t_*, and note that the probability of success is simply *(1−p_t_)*. A survival time of *r* for a module of size *c* born at step *b* then implies there were *(r−1)* successes at steps *b+1, b+2,…, b+r−1* followed by a failure at step *b+r*. The probability of failure at step *b+j* can be computed as follows: there are *N−(b+j)* edges remaining, of which *c (n−c)* constitute failures. Therefore the probability of failure at step *b+j* is *p_b+j_ = c (n−c)/(N−b−j)*. The null distribution of the survival time *S* is given by
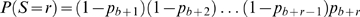
We approximate these probabilities by setting *p_b+j_* to the constant *p_b+1_*, leading to an approximating geometric distribution for *S*. Ling established that this approximation is good if *r* is small compared to *N−b*.

### Generalized isolation

To address the problem of mistimed edges, we consider a more general set of survival times: the waiting times to the 1st, 2nd ,…, *k*-th failure, where *k* is a low number (we evaluated up to *k* = 6).

To derive the null distribution of these survival times, we can use the same reasoning, except that the number of the number of edges that lead to failure is now *c (n−c)−k−1* so that *p_b+j_ = (c (n−c)−k−1)/(N−b−j)*. We are now waiting for the *k*-th failure, so the approximating distribution is negative binomial rather than geometric. These two distributions are identical for *k = 1*.

The situation where a group of genes is hit before birth requires a different approach. To identify such groups, we analyze a modified graph process: we remove all *n−1* module-killing edges individually from the original graph process and apply our method to the modified process, leading to the Miso(1,*k*) method. Iterating this procedure by removing all *n−1* module-killing edges from all *n−1* modified processes leads to the Miso(2,*k*) method. For any candidate module, we utilize the minimum p-value for 1, 2, …, *k*.

### Analyses of yeast mutant phenotype data

In all methods, we did not consider groups of size greater than 50 to be valid candidate modules since the true biological modules of interest are of much smaller size. For the Miso method, the results did not vary noticeably for a wide range of size cutoffs.

For the MCL method, we picked a sequence of threshold graphs with 500, 1000, … up to half of the relationships available, at which point MCL only finds a few clusters. We also tried different settings of the granularity tuning parameter for MCL but found that while this parameter can improve the results for a single graph, the set of results for our sequence of graphs did not benefit from choosing different levels of granularity. For this reason we ran each MCL procedure with the default granularity setting.

For the global cuts of dendrograms, we chose cuts leading to 3, 10, 20, …, 100 clusters. We found that cuts leading to few clusters did not perform well, but included 3 since it was the value chosen by the gap statistic [17]. To create Figure 4 and Figure S1, we retrieved the height corresponding to the chosen number of clusters and matched it to the rank in the process (number of edges with similarity less or equal to this height)

In contrast to the gap statistic and other methods that can be applied to any partitioning (from a hierarchical or any other clustering), local cuts (sometimes called “stopping rules”) are restricted to hierarchical agglomerative methods. The cuts are conducted as formal tests if the cluster resulting from a merge step in the clustering contains one or two clusters (the joins are performed until there is evidence against the null-hypothesis of one cluster). For any chosen rejection value for the test statistic, the method delivers a set of candidate modules. To perform this test, assumptions about the distribution of the data have to be made, given in detail in [19].

All clustering except for MCL (version 06-058, default settings) was done in R [13]. R code and a Cytoscape plug-in are available from http://www.stat.ubc.ca/jenny/webSupp/brummMiso/.

## Supporting Information

Figure S1 Relative performance of module detection methods applied to yeast DNA damage response data. Displayed are the PPV (top row) and sensitivity (bottom row). The horizontal axes correspond to the tuning parameters specific to each class of methods; see Materials and methods. For the Miso methods in column a), the tuning parameter is the threshold applied to module-specific p-values., Ffor the local cuts in column b) the tuning parameter is the rejection value for the Duda-Hart test statistic. For the global methods in column c), the tuning parameter corresponds to a step in the graph process. Column d) summarizes the range of PPV and sensitivity values.
(0.22 MB TIF)
