# Supplementary figures and images for: Discovery and Expansion of Gene Modules by Seeking Isolated Groups in a Random Graph Process
Source: PLoS One. 2008 Oct 9;3(10):e3358. doi: 10.1371/journal.pone.0003358 (PMC2559867; doi:10.1371/journal.pone.0003358)

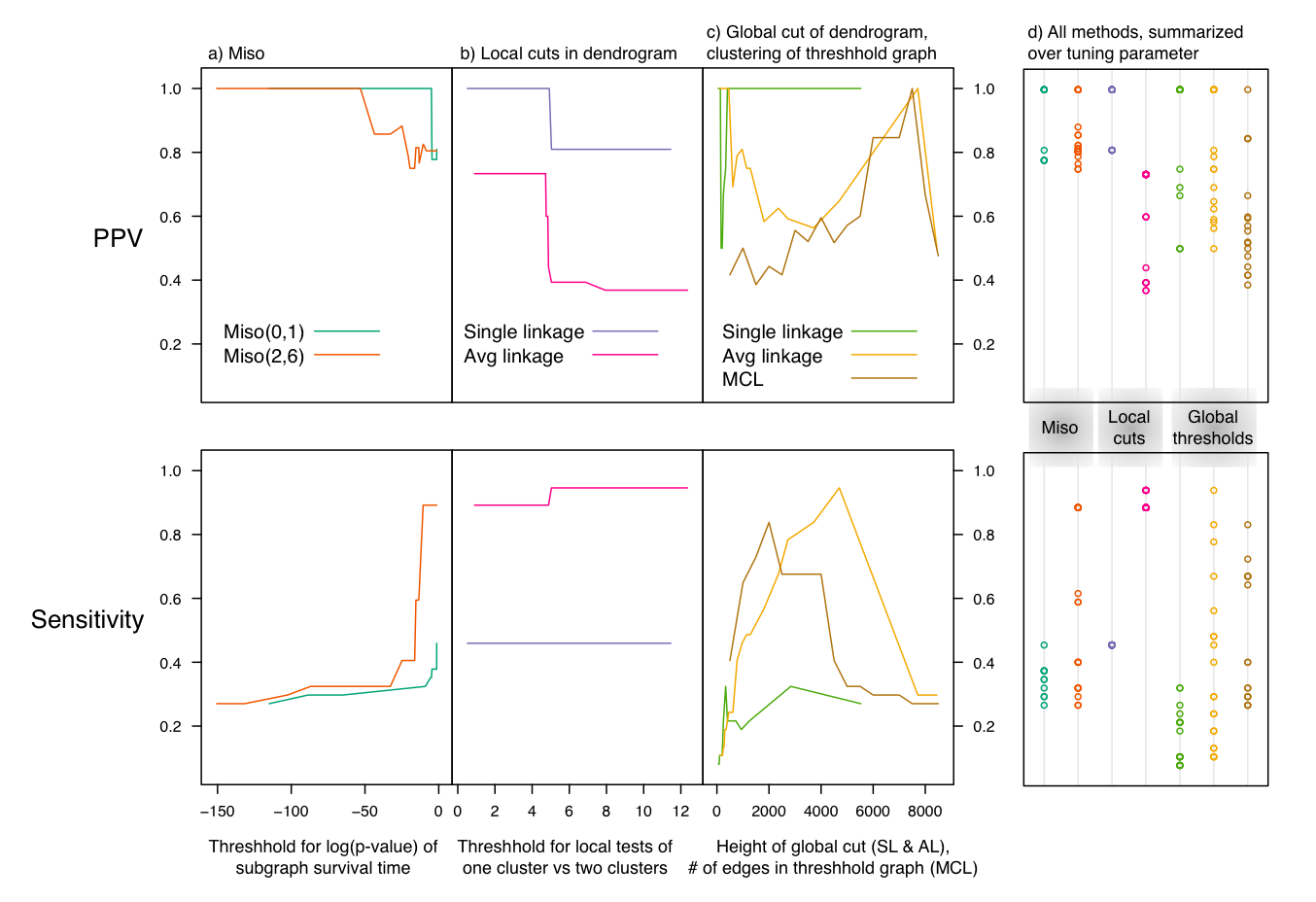

Supplement: Figure S1 — Relative performance of module detection methods applied to yeast DNA damage response data. Displayed are the PPV (top row) and sensitivity (bottom row). The horizontal axes correspond to the tuning parameters specific to each class of methods; see Materials and methods. For the Miso methods in column a), the tuning parameter is the threshold applied to module-specific p-values., Ffor the local cuts in column b) the tuning parameter is the rejection value for the Duda-Hart test statistic. For the global methods in column c), the tuning parameter corresponds to a step in the graph process. Column d) summarizes the range of PPV and sensitivity values. (0.22 MB TIF) [file pone.0003358.s001.tif]
